# Activation of the Hedgehog pathway mediates resistance to epidermal growth factor receptor inhibitors in non-small cell lung cancer

**DOI:** 10.7150/jca.63410

**Published:** 2022-01-04

**Authors:** Hualin Chen, Donghong Yang, Yongcun Wang, Hua Tao, Yiping Luo, Aibing Wu, Shujun Li, Zhixiong Yang, Ming Chen

**Affiliations:** 1 Department of Pulmonary Oncology, Affiliated Hospital of Guangdong Medical University, Zhanjiang 524023, China; 2 Department of Oncology, Affiliated Hospital of Guangdong Medical University, Zhanjiang 524023, China; 3 Department of Radiotherapy, Jiangsu Cancer Hospital, Jiangsu Institute of Cancer Research, Nanjing Medical University Affiliated Cancer Hospital, Nanjing, Jiangsu 210009, China; 4 Institute of Cancer Research and Basic Medical Sciences of Chinese Academy of Sciences, Cancer Hospital of University of Chinese Academy of Sciences, Hangzhou 310011, China; 5 Department of Radiation Oncology, Zhejiang Key Lab of Radiation Oncology, Zhejiang Cancer Hospital, Hangzhou 310011, China

**Keywords:** Sonic Hedgehog Protein, Tyrosine Kinase Inhibitors, Epithelial-Mesenchymal Transition, Non-Small Cell Lung Cancer, Zinc Finger Protein GLI1

## Abstract

The current study aimed to investigate the function of the Hedgehog pathway and its association with epithelial-mesenchymal transition (EMT) in epidermal growth factor receptor (EGFR) tyrosine kinase inhibitor (TKI) resistance in non-small cell lung cancer (NSCLC). Lung tumor tissue specimens from EGFR TKI-resistant patients, including those with brain metastases, had hyperactive Hedgehog signaling compared with those from TKI-sensitive patients. SHH stimulation promoted GLI1 activation as well as cell motility in parental PC9 cells while suppressing gefitinib-induced apoptosis in gefitinib-resistant cells. SHH also promoted EMT in parental PC9 cells via E-cadherin suppression and N-cadherin and vimentin upregulation. The knockdown of GLI1 exhibited the opposite effects. Besides, SHH induced, whereas GLI1 knockdown reversed gefitinib resistance in xenograft tumors. The Hedgehog pathway inhibitor GDC-0449 synergized with gefitinib to increase xenograft tumor sensitivity to chemotherapy and extend survival in tumor-bearing animals. These results suggest the Hedgehog pathway mediates EGFR TKI resistance and induces EMT in NSCLC, representing a potential therapeutic target to defeat TKI resistance.

## Introduction

Lung cancer remains the commonest and deadliest malignancy around the world 1. Non-small cell lung cancer (NSCLC) comprises about 85% of all cases of lung cancer 2. Brain metastases are found in approximately 10% of NSCLC cases at initial diagnosis and occur in 25%-40% during the course of the disease 3. Despite the development of systemic therapies combined with radiotherapy, NSCLC with brain metastases remains incurable, and patients survive only four months on average 4.

EGFR mutations are detected in 20%-40% of NSCLC patients and promote cancer progression. Exon 19 deletions and exon 21 L858R substitution amount to approximately 80%-90% of all mutated EGFR 5. Although most NSCLC cases harboring EGFR mutations respond to initial therapy with first-generation tyrosine kinase inhibitors (TKIs), including erlotinib and gefitinib 6, 7, many of them develop TKI resistance within a year after the initiation of treatment 8. Understanding the mechanisms of EGFR TKI resistance could help overcome TKI resistance in NSCLC.

Acquired T790M substitution and MET amplification constitute two primary mechanistic events responsible for EGFR TKI resistance in NSCLC. Epithelial-mesenchymal transition (EMT) and Hedgehog pathway induction also contribute to TKI tolerance 9. EMT is marked by epithelial cells losing cell junction proteins such as E-cadherin and Zonula occludens-1 protein (ZO-1) and gaining mesenchymal biomarkers such as N-cadherin and vimentin, leading to a metastatic switch^10^. EMT-type lung cancer cells are tolerant to EGFR TKIs, and reverting EMT improves the efficacy of TKIs 11, 12. Hedgehog signaling contains three hedgehog homologs (Sonic, Indian, and desert hedgehogs), the transmembrane receptor Patched (PTCH), the cell surface molecule Smoothened (SMO), and the transcription factor glioma-associated oncogene homolog 1 (GLI1) 13. The primary ligand Sonic hedgehog (SHH) initiates the Hedgehog pathway by binding to PTCH, allowing SMO to release from PTCH-mediated suppression. Following SMO activation, GLI1 is released from the fused suppressor and translocated to the nucleus, thereby inducing transcription of downstream genes controlling cell survival, EMT, and stem cell self-renewal 14.

Recent studies demonstrated that Hedgehog signaling mediates EMT in acquired EGFR TKI resistance. Meanwhile, blocking Hedgehog signaling reverses EMT to enhance the sensitivity of NSCLC cells to TKIs 15, 16. Here, the Hedgehog pathway was investigated for its function in EGFR TKI resistance. In addition, we evaluated the potential therapeutic benefits of blocking Hedgehog signaling to overcome TKI-resistant NSCLC.

## Materials and Methods

### Cell culture

The PC-9 (Ex19del) cell line is a common EGFR-sensitive adenocarcinoma cell line used in many studies. A previous study showed that hedgehog was activated in HCC827 cells 16. Human PC9 NSCLC cells were provided by American Type Culture Collection (USA) and maintained in McCoy's 5A medium (Gibco, USA) supplemented with 10% fetal bovine serum (FBS; Gibco) in an incubator with 5% CO_2_. Gefitinib-resistant PC9 cells were derived by culturing parental PC9 cells in 10 μmol/L gefitinib (AstraZeneca, UK) for 48 h, followed by treatment with 0.01-10 μmol/L gefitinib for eight months. The drug was refreshed every 72-96 h. Gefitinib-resistant PC9 cells were cultured in 0.5 µmol/L gefitinib to maintain resistance.

### Patients and specimens

This study recruited 31 NSCLC cases showing brain metastases who were admitted to the Department of Pulmonary Medicine at the Affiliated Hospital of Guangdong Medical University (Guangdong, China) between January 2016 and February 2018. Patient baseline features are summarized in Table 1. Inclusion criteria were: (1) histopathologically-proven primary NSCLC; (2) CT- or MRI-confirmed brain metastases; (3) ≥ 1 measurable extracranial or intracranial lesions; (4) carrying exon 19 deletion and/or exon 21 L858R substitution confirmed by amplification refractory mutation system 17; (5) Eastern Cooperative Oncology Group performance status 18 = 0-2; (6) no history of chemotherapeutic or radiation use to treat brain metastasis; (7) no other malignancies; (8) treatment with a first-generation EGFR-TKI as the initial anticancer therapy. Patients with incomplete clinical or follow-up data were excluded. The therapeutic responses were evaluated following The Response Evaluation Criteria in Solid Tumors 18. According to the Jackman criteria, 11 patients who responded to initial targeted treatment and developed disease progression six months later were considered having acquired TKI resistance 19. The remaining 20 patients were classified as TKI-sensitive. Tumor specimens were acquired by percutaneous lung biopsy or thoracic surgery.

This trial had approval from the Ethics Committee of The Affiliated Hospital of Guangdong Medical University (Guangdong, China), and the procedures complied with the Good Clinical Practice Guidelines and the 1975 Declaration of Helsinki. All patients provided signed informed consent.

### Generation of lentiviral vectors and transfection

Small hairpin RNA (shRNA) against GLI1 (shGLI1) was cloned into pLKO.1 lentiviral vector (Addgene, USA). The shRNA sequences are shown in Table S1. Gefitinib-resistant PC9 cells were transduced with empty and shRNA-GLI1-overexpressing vectors, respectively. All subsequent assays were performed 48 h after transduction.

### Tumor xenograft

Ten BALB/c nude mice (5-week old) provided by Shanghai Silaike Experiment Animal Co. (China) were housed in a specific pathogen-free facility. Experiments involving animals had approval from the Ethics Committee of Guangdong Medical University and followed the Guidelines for Proper Conduct of Animal Experiments of Guangdong Medical University.

To examine the role of SHH stimulation, we divided the animals into control and SHH groups (n = 6/group). Then, 2 × 10^6^ PC9 cells in 100 µL phosphate-buffered saline (PBS) or SHH (0.5 µg/mL)-containing PBS were inoculated subcutaneously into both armpits of each mouse. To investigate the role of GLI1 knockdown, mice were divided into three groups (n = 6/group) first. Then, 2 × 10^6^ TKI-resistant PC9 cells overexpressing control shRNA, shGLI1, or shGLI2 were resuspended in 100 µL PBS and inoculated by subcutaneous injection into both animal armpits. Tumor formation was monitored every other day. Upon reaching 5 mm in diameter, the tumor was measured every week with a digital caliper. Tumor volume (V) was derived as (length×width^2^)/2. With tumors measuring 8 mm in diameter, the animals underwent treatment with gefitinib (20 mg/kg) five consecutive days per week for six weeks by gavage. Anesthesia was performed using pentobarbital, 40-50 mg/kg IP. The mice were sacrificed by cervical dislocation, decapitation, exsanguination, and bilateral pneumothorax. The tumors were immediately removed, weighed, and stored at -80°C.

To assess the synergistic effects of the TKI and hedgehog inhibitor on TKI-resistant tumors, we inoculated the animals with TKI-resistant PC9 cells. When tumor diameter reached 8 mm, the animals were administered control, gefitinib (20 mg/kg), GDC-0449 (32 mg/kg), or gefitinib + GDC-0449 five consecutive days per week for six weeks by gavage. Tumor volumes and survival of tumor-bearing mice were recorded.

### Sample collection from the TCGA database

The data of reverse-phase protein arrays (RPPA), overall survival (OS), and progression-free survival (PFS) were collected from the TCGA database.

### SHH treatment

PBS with 0.1% bovine serum albumin (BSA) was employed to dissolve SHH (R&D System, USA). Parental PC9 cells underwent stimulation with SHH (0.5 μg/mL) for 24 or 48 h before further experiments.

### Quantitative real-time PCR

Total RNA extraction was carried out with TRIzol (Fisher Scientific), and reverse transcription utilized a PrimeScriptTMRT Master Mix (TAKARA, Japan). Amplification was performed using gene-specific primers (Table S2) and FastStart Universal SYBR Green Master Mix on a GeneAmp^®^ StepOne Real-Time PCR System (Fisher Scientific). The 2^-ΔΔCt^ method was applied for data analysis, with GAPDH employed for normalization.

### Immunoblot

PC9 cells or tumor tissues underwent lysis or homogenization using RIPA buffer. Lysate samples were centrifuged (12,000 *g*, 15 min), and the supernatants were collected. Equal amounts of total protein (Bradford method) underwent separation by 10% SDS-PAGE and were transferred onto polyvinylidene difluoride (PVDF) membranes. TBST with 5% fat-free milk was employed to block the membranes (1 h), which underwent incubation with the primary antibodies (1:1000) raised against SMO (Abcam, UK) and GLI1, SNAIL, E-cadherin, N-cadherin, vimentin, and β-actin (Cell Signaling, USA), respectively, overnight at 4°C. This was followed by treatment with horseradish peroxidase (HRP)-linked secondary antibodies for 1 h at room temperature. Chemiluminescent signals were detected on X-ray films and quantified with the Lab Works software (Lehi, UT, USA).

### Cell migration and invasion assays

For cell migration evaluation, 3×10^4^ cells were plated in the upper part of the Transwell device (8-μm pore size). For cell invasion assay, Matrigel-coating of the upper chamber was performed before cell plating in serum-free medium. The specimens were incubated for 24 h at 37°C, and nonmigrating or non-invading cells remaining in upper chambers were wiped with cotton swabs. The migrating or invading cells adhering to the lower surface underwent fixation and crystal violet (0.2%) staining. A Nikon inverted light microscope (Japan) was utilized to count stained cells in five high-power random fields at 200× magnification. Images were acquired using a Nikon camera.

### Flow cytometry

Cell apoptosis assessment was performed with an Annexin -V-FLUOS staining kit (Roche, Switzerland) as directed by the manufacturer. In brief, PC9 cells underwent washes with PBS and were harvested by trypsinization. Annexin V and propidium iodide were added for 15 min away from light. The analysis was carried out on a FACSCalibur flow cytometer (BD Biosciences, USA). The data were quantified using CXP 1.0 (Beckman Coulter, USA).

### Immunofluorescent staining

PC9 cells were fixed with polybutylene terephthalate and underwent permeabilization (10 min) with 0.5% Triton X-100 (Sigma) in PBS. After blocking in PBS with 2% BSA (30 min), cells were incubated for 2 h with primary antibodies (1:100) targeting E-cadherin, ZO1, N-cadherin, and vimentin (Cell Signaling), respectively, at 4°C. This was followed by incubation with Alexa Fluor 488 for 2 h at ambient. DAPI was employed for counterstaining, and samples were mounted in a mounting medium for fluorescent imaging. Images were acquired under an Eclipse TE2000-U immunofluorescence microscope (Nikon, Japan).

### Immunohistochemical staining

After paraffin-embedding, the blocks were sectioned at 5 μm. The sections were deparaffinized and rehydrated, followed by immunohistochemical staining. Briefly, after blocking with FBS for 10 min, the samples underwent overnight incubation with primary antibodies (1:100) targeting SMO (Abcam), and GLI1, SNAIL, and BCL2 (Cell Signaling), respectively, at 4℃. After PBS washes, the sections underwent incubation with an HRP-linked secondary antibody (Cell Signaling) at ambient for an additional 30 min. Normal IgG was employed as a negative control. An inverted fluorescence microscope (Nikon) was used for visualization, and brown staining was considered positive.

### Statistical analysis

Continuous data are presented as means ± standard deviation (SD) and were analyzed using Student's t-test and ANOVA with the LSD post hoc test. Categorical variables were presented as frequency or percentage of the total, and statistical analyses were carried out with SPSS 21.0 (SPSS, USA). The *X^2^* test was performed for group pair comparisons. The correlation between GLI1 expression and the survival time of patients was assessed using Kaplan-Meier analysis and the log-rank test. *P*<0.05 indicated statistical significance.

## Results

### Hedgehog signaling-related genes are overexpressed in NSCLC with EGFR TKI resistance

**Figure 1:**
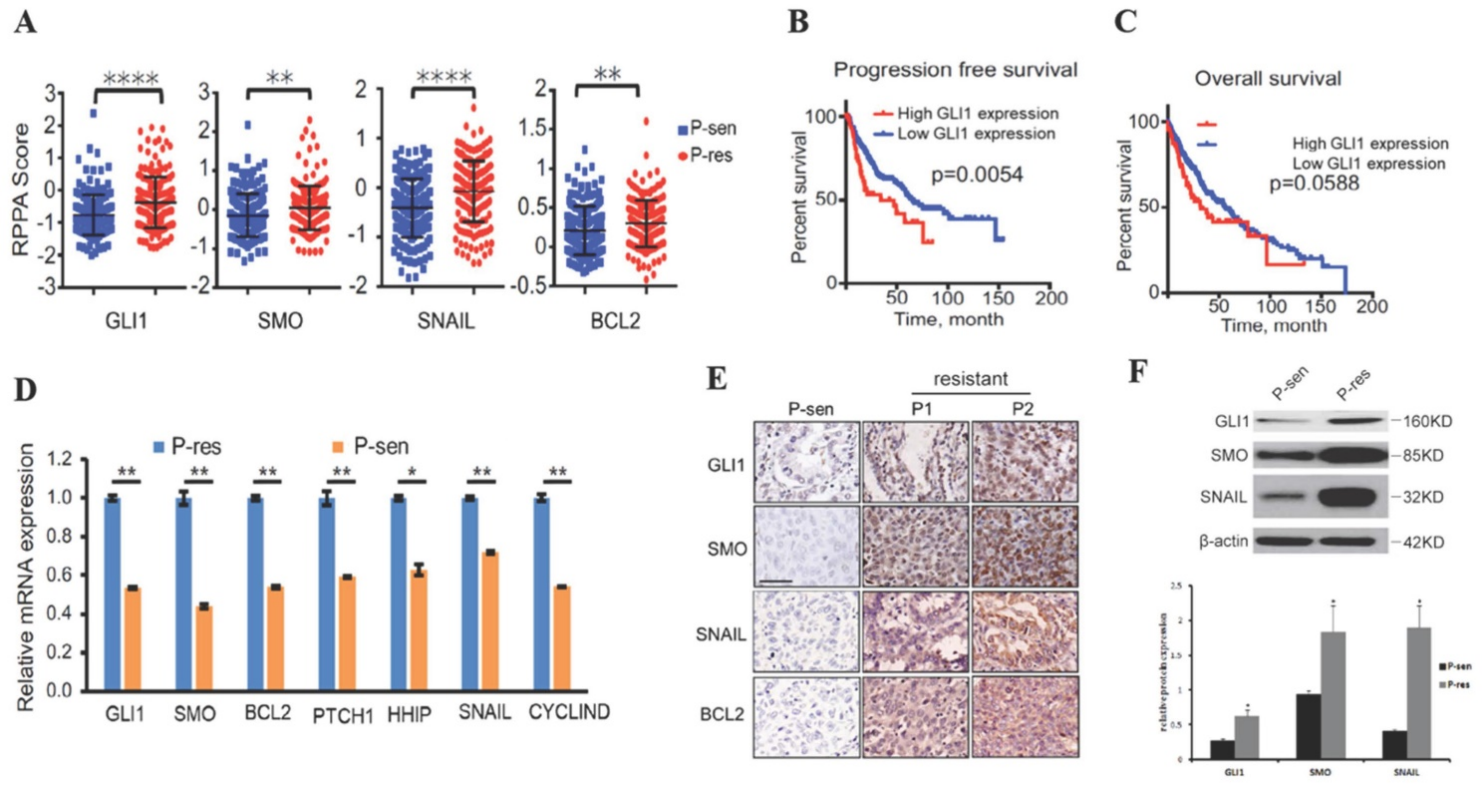
**Hedgehog pathway components are overexpressed in EGFR TKI-resistant NSCLC.** (A) Reverse-phase protein arrays (RPPA) data for GLI1, SMO, SNAIL, and BCL2 were collected from The Cancer Genome Atlas (TCGA) database. Continuous data are presented as means ± standard deviation (SD) and were analyzed using Student's t-test. (B and C) The associations of GLI1 expression with progression-free survival and overall survival (collected from TCGA) were analyzed by the Kaplan-Meier (KM) method and log-rank test. (D) According to the Jackman criteria, 31 NSCLC patients with brain metastasis were recruited and divided into EGFR TKI-resistant (n = 11) and -sensitive (n = 20) groups. Quantitative real-time PCR (qRT-PCR) was performed to determine the mRNA expression levels of GLI1, SMO, BCL2, PTCH1, HHIP, SNAIL, and CYCLIND in lung cancer tissue samples from the patients. Continuous data are presented as means ± standard deviation (SD) and were analyzed using Student's t-test. **P<0.01 vs. P-sen. Immunofluorescent staining (E) and Western blot analysis (F) were performed to determine the protein expression levels of GLI1, SMO, and SNAIL in lung cancer tissue specimens. Scale bar = 20 µm. Continuous data are presented as means ± standard deviation (SD) and were analyzed using Student's t-test. *P<0.05 vs. P-sen. GLI1, glioma-associated oncogene homolog 1; SMO, smoothened; BCL2, B-cell lymphoma 2; PTCH1, Patched; HHIP, Hedgehog interacting protein; P, patient; sen, sensitive; res, resistant.

To examine whether the Hedgehog pathway is linked to EGFR TKI resistance in NSCLC, we collected the RPPA data, OS, and PFS of NSCLC cases from the TCGA database. The results showed that tumor tissues from EGFR TKI-resistant patients had significantly increased GLI1, SMO, BCL2, and SNAIL protein levels compared with those from TKI-sensitive cases (Fig. 1A), suggesting that these proteins are possibly related to TKI resistance in NSCLC. Patients with higher GLI1 expression had shorter PFS (*P*=0.0054) and OS (*P*=0.0588) compared with those showing lower GLI1 expression (Fig. 1B and 1C), suggesting that GLI1 overexpression predicts a poorer prognosis in NSCLC.

We further assessed the alterations of hedgehog signaling in EGFR TKI resistance in NSCLC cases showing brain metastases. Interestingly, qRT-PCR, immunohistochemical staining, and Western blot analysis consistently demonstrated that tumor tissue samples from TKI-resistant patients had remarkably increased mRNA and protein expression of hedgehog signaling associated components compared with those from TKI-sensitive patients (Fig. 1 D-F). These findings suggested that activated Hedgehog signaling was also associated with EGFR TKI resistance in NSCLC cases showing brain metastases.

### Activation of Hedgehog signaling promotes cell mobility while suppressing gefitinib-induced apoptosis in parental PC9 cells

**Figure 2:**
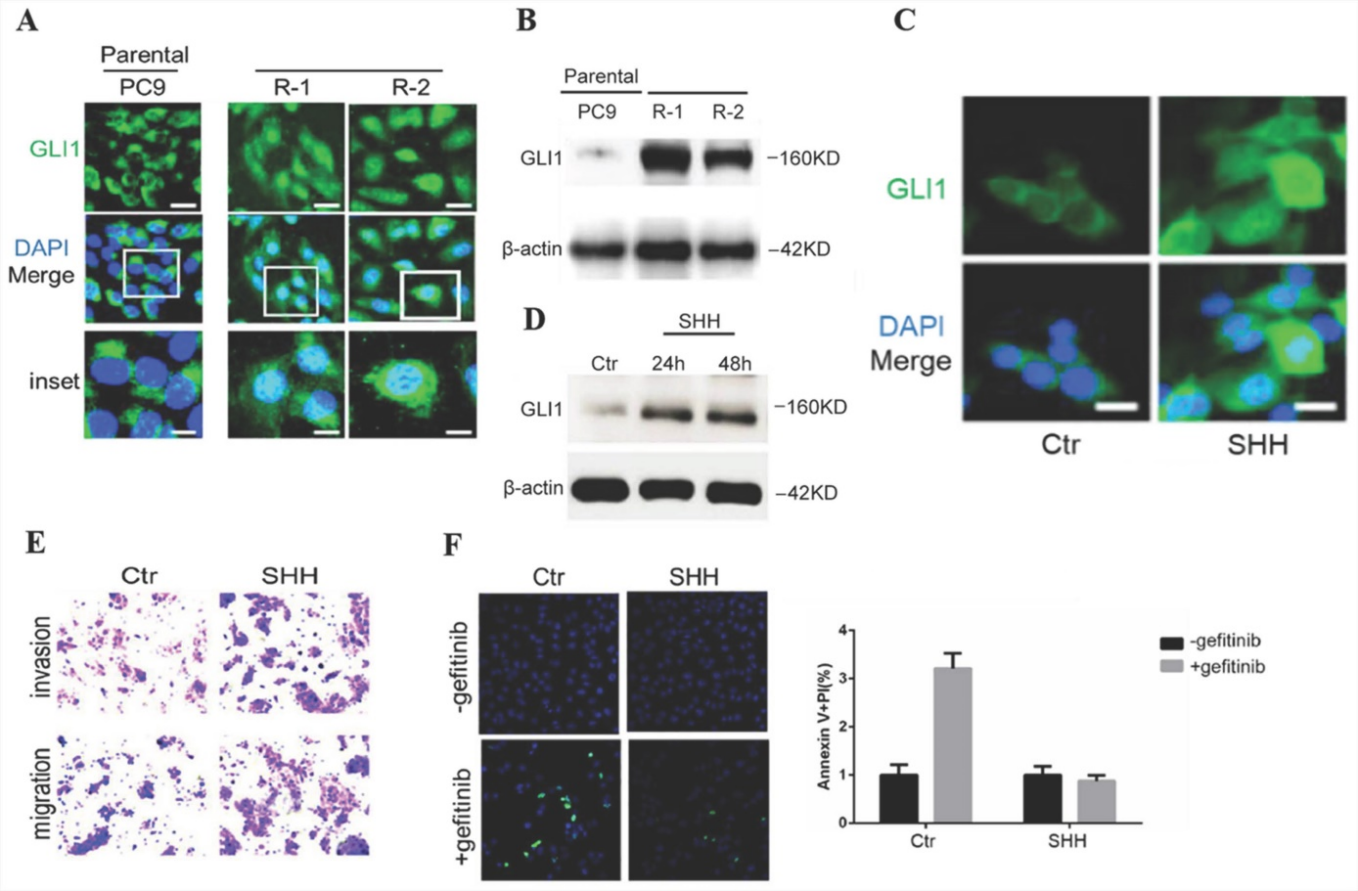
**SHH stimulation promotes cell motility while suppressing gefitinib-induced apoptosis in parental PC9 cells.** (A and B) Immunofluorescent staining and Western blot analysis were performed to detect endogenous GLI1 protein expression in parental and gefitinib-resistant PC9 cells. (C and D) Parental PC9 cells were treated with recombinant Sonic hedgehog (SHH; 0.5 μg/mL) for 24 h or 48 h. GLI1 expression was detected by immunofluorescent staining and Western blot analysis. (E) Cell migration and invasion assays. (F) Cell apoptosis was evaluated by immunofluorescent staining with FITC-labeled annexin V (green) followed by flow cytometry. Continuous data are presented as means ± standard deviation (SD) and were analyzed using Student's t-test. N = 3. Scale bar = 20 µm. GLI1, glioma-associated oncogene homolog 1; R, resistant; Ctr, control; SHH, sonic hedgehog.

To investigate Hedgehog signaling's involvement in EGFR TKI resistance in NSCLC, gefitinib-resistant PC9 cells were generated from parental sensitive cells and assessed for endogenous amounts of GLI1. Compared with parental cells, gefitinib-resistant PC9 cells demonstrated dramatically enhanced GLI1 protein amounts and nuclear accumulation (Fig. 2A and 2B), indicating that Hedgehog signaling was activated in gefitinib-resistant PC9 cells. The overexpression of GL1 is not temporary since it remains high even after replacing the culture medium with a medium without gefitinib. SHH treatment induced GLI1 translocation into the nucleus as well as a considerable elevation of GLI1 protein levels in parental PC9 cells (Fig. 2C and 2D). SHH stimulation also enhanced cell migration and invasion, inhibiting gefitinib-induced apoptosis in parental PC9 cells (Fig. 2E and 2F). The above data suggested that SHH/GLI1 signaling activation promoted motility in PC9 cells while attenuating their sensitivity to gefitinib.

### The knockdown of GLI1 inhibits the motility of gefitinib-resistant PC9 cells while promoting gefitinib-induced apoptosis

**Figure 3:**
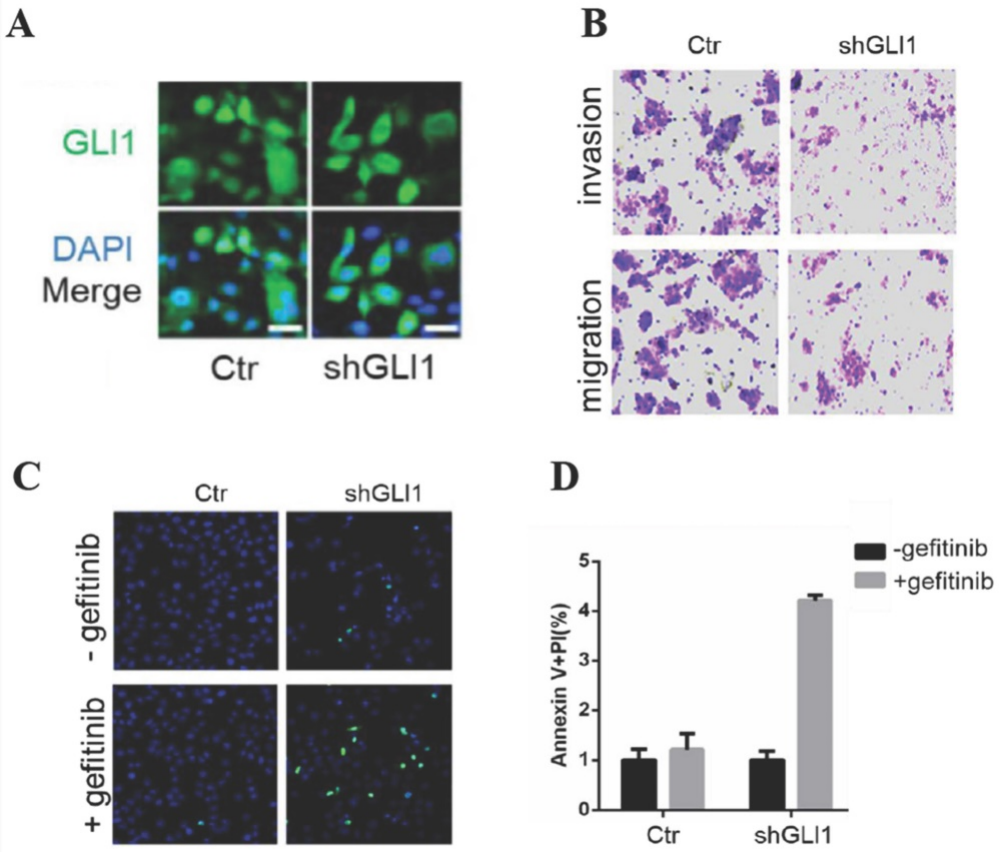
** The knockdown of GLI1 inhibits cell motility while promoting gefitinib-induced apoptosis in gefitinib-resistant PC9 cells.** Gefitinib-resistant PC9 cells were transfected with a lentiviral vector expressing small hairpin RNA against GLI1 (shGLI1) or control for 48h. (A) The knockdown efficacy of GLI1 was assessed by immunofluorescent staining. (B) Cell migration and invasion assays. (C and D) Cell apoptosis was evaluated by immunofluorescent staining with FITC-labeled annexin V (green) followed by flow cytometry. Continuous data are presented as means ± standard deviation (SD) and were analyzed using Student's t-test. N = 3. Scale bar = 20 µm. GLI1, glioma-associated oncogene homolog 1; R, resistant; Ctr, control; shGLI1, small hairpin RNA of GLI1.

Next, we examined whether targeting GLI1 could reverse PC9 cell resistance to gefitinib. The results (Fig. 3A and 3B) revealed that shGLI1-induced silencing of GLI1 remarkably suppressed cell migration and invasion in gefitinib-resistant PC9 cells. The knockdown of GLI1 also dramatically promoted apoptosis in gefitinib-resistant PC9 cells (Fig. 3B-D). These findings suggested that knockdown of GLI1 overcame EGFR TKI resistance in PC9 cells.

### SHH stimulation or GLI1 knockdown regulates EMT markers

**Figure 4:**
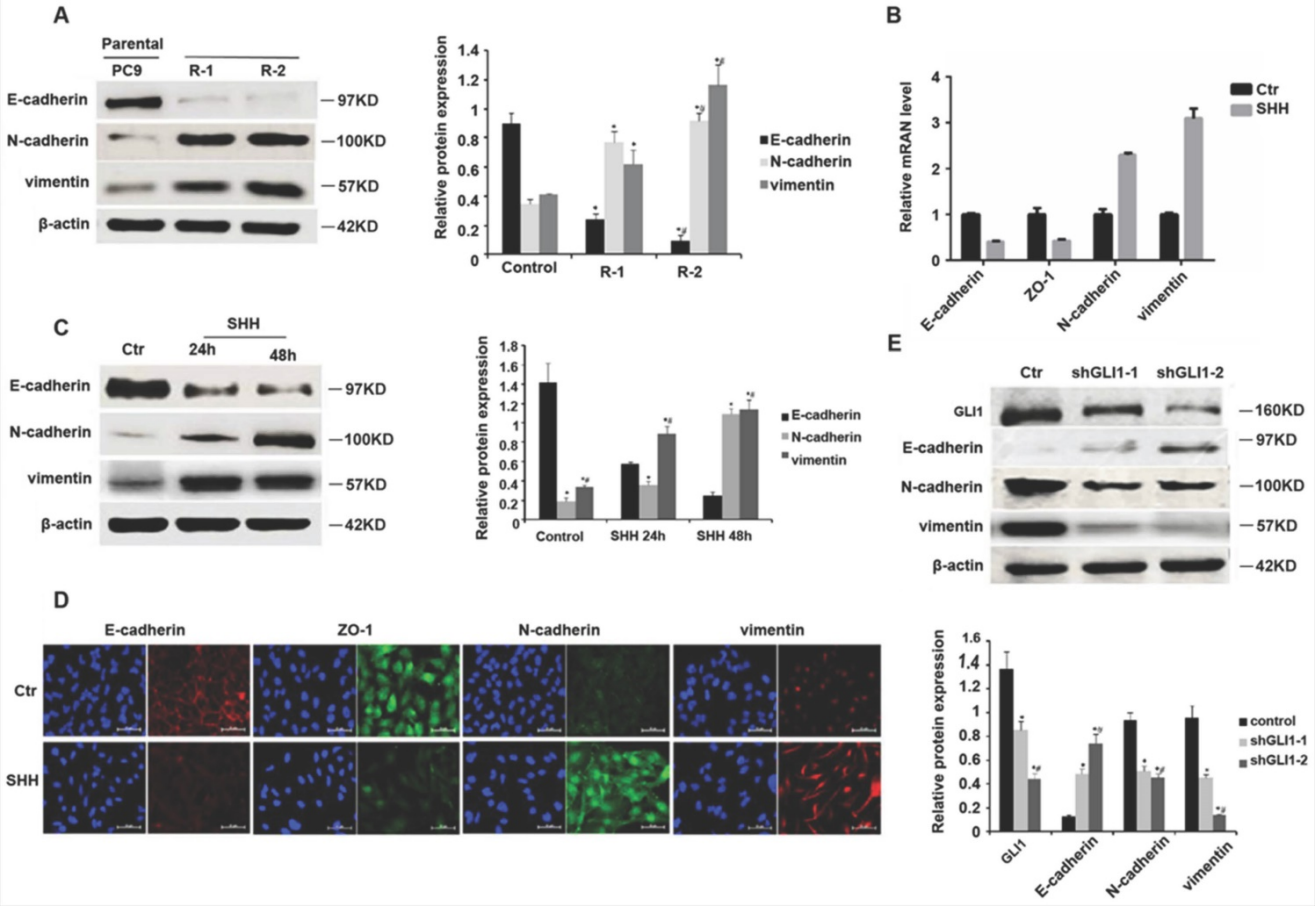
** SHH stimulation or GLI1 knockdown regulates EMT markers.** (A) Western blot analysis was performed to determine the protein expression levels of E-cadherin, N-cadherin, and vimentin in parental and gefitinib-resistant PC9 cells. Continuous data are presented as means ± standard deviation (SD) and were analyzed using ANOVA and LSD post hoc test. *P<0.05 vs parental PC9 cells; #*P*<0.05 vs. R-1. N = 3. Parental PC9 cells were treated with recombinant Sonic hedgehog (SHH; 0.5 μg/mL) for 24 h or 48 h. (B) Quantitative real-time PCR (qRT-PCR) was performed to determine the mRNA expression levels of E-cadherin, ZO-1, N-cadherin, and vimentin. (C) Western blot analysis was performed to determine the protein expression levels of E-cadherin, N-cadherin, and vimentin. Continuous data are presented as means ± standard deviation (SD) and were analyzed using ANOVA and LSD post hoc test. *P<0.05 vs control; #*P*<0.05 vs. 24 h. N = 3. Gefitinib-resistant PC9 cells were transfected with lentiviral vectors expressing shGLI1 and control shRNA, respectively. At 48h after transfection, (D) immunofluorescent staining was performed to detect the protein expression levels of E-cadherin, ZO-1, N-cadherin, and vimentin; (E) Western blot analysis was performed to determine the protein expression levels of E-cadherin, N-cadherin, and vimentin. Continuous data are presented as means ± standard deviation (SD) and were analyzed using ANOVA and LSD post hoc test. *P<0.05 vs control; #*P*<0.05 vs. shGLI1-1. N = 3. Scale bar = 20 µm. GLI1, glioma-associated oncogene homolog 1; R, resistant; Ctr, control; SHH, sonic hedgehog; shGLI1, small hairpin RNA of GLI1.

EMT contributes to EGFR TKI resistance in NSCLC 20, 21. GLI1 amounts show negative associations with EMT marker expression levels 22. Therefore, we assessed whether SHH/GLI1 signaling could regulate EMT in PC9 cells. As shown in Fig. 4A, gefitinib-resistant PC9 cells had significantly reduced E-cadherin protein amounts while increasing N-cadherin and vimentin protein levels, compared with the parental cells, indicating an involvement of EMT in gefitinib resistance. SHH stimulation promoted EMT in parental PC9 cells, as evidenced by E-cadherin and ZO-1 downregulation and N-cadherin and vimentin upregulation (Fig. 4B and 4C). GLI1 knockdown exhibited opposite effects in gefitinib-resistant PC9 cells (Fig. 4D and 4E). These findings indicated that Hedgehog pathway activation was linked to EMT induction in EGFR TKI resistance.

### SHH induces, whereas GLI1 knockdown reverses gefitinib resistance in xenograft tumors

**Figure 5:**
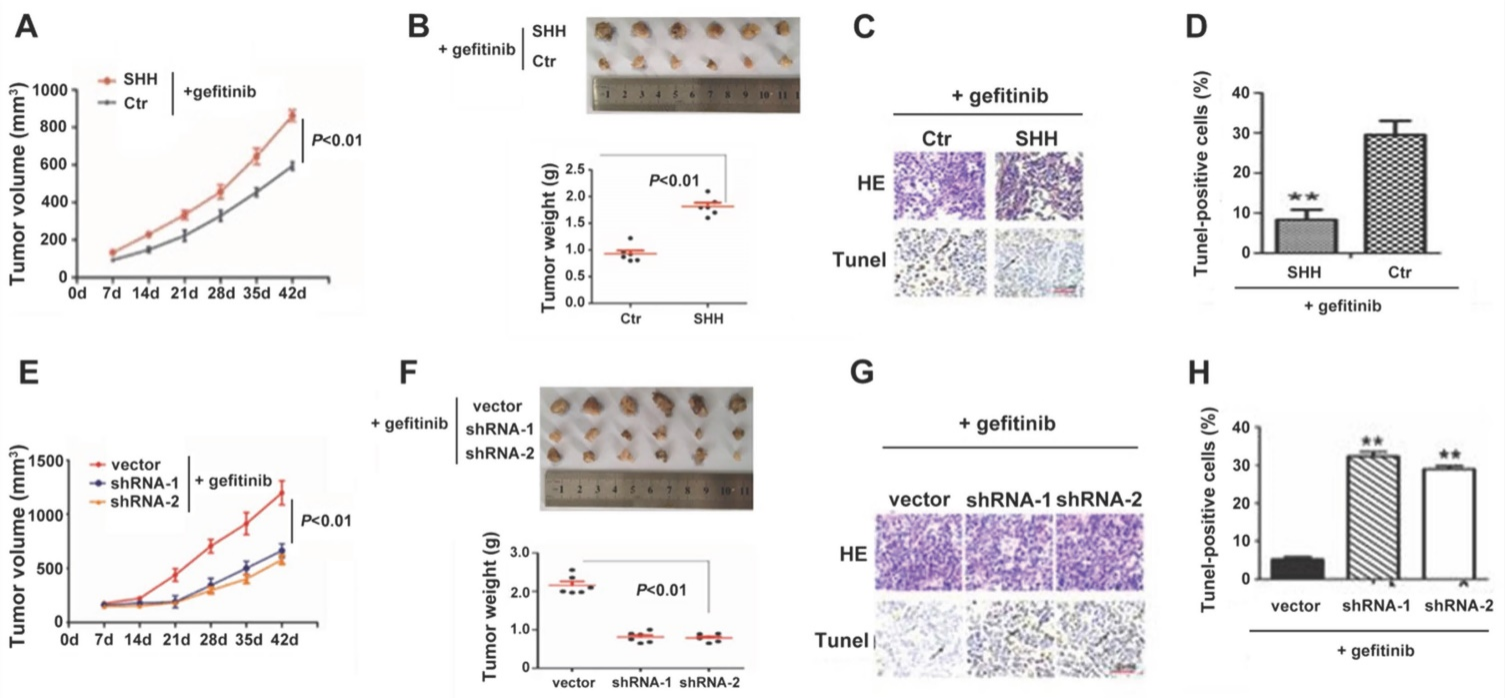
** SHH induces, whereas GLI1 knockdown reverses gefitinib resistance in xenograft tumors.** (A - D) BALB/c nude mice (5-week-old) were randomly divided into control and SHH groups (n = 6/group). Then, 2 × 10^6^ parental PC9 cells were resuspended in 100 µL PBS or PBS containing SHH (0.5 µg/mL) and inoculated subcutaneously into both armpits of each mouse. (E - H) Mice were randomly divided into three groups (n = 6/group). Gefitinib-resistant PC9 cells (2 × 10^6^) transfected with empty vector, shGLI1, or shGLI2 were resuspended in 100 µL PBS and inoculated subcutaneously into both armpits of each mouse. (A and E) After the tumor reached 5 mm in diameter, tumor volume (V) was measured every week and derived as V=(length×width^2^)/2. After the tumor reached 8 mm in diameter, gefitinib (20 mg/kg) was administered five consecutive days per week for six weeks by gavage. (B and F) Mice were sacrificed, and the tumors were immediately removed and weighed. (C and G) Tumor tissue specimens were subjected to hematoxylin and eosin (H&E) staining and TUNEL assay. Representative images are shown. Scale bar = XXX. (D and H) The percentages of TUNEL-positive cells were calculated. Continuous data are presented as means ± standard deviation (SD) and were analyzed using ANOVA and LSD post hoc test. **P<0.01 vs. control or empty vector. N = 3. SHH, sonic hedgehog; Ctr, control; shGLI1, small hairpin RNA of GLI1; HE, hematoxylin, and eosin.

We then established a nude mouse xenograft model for examining Hedgehog signaling's function in TKI resistance *in vivo*. As shown in Fig. 5A and 5B, SHH-treated PC9 cells generated significantly greater tumor masses than control cells under gefitinib treatment. The TUNEL assay revealed that SHH exposure significantly suppressed tumor cell apoptosis (Fig. 5C and 5D). These results indicated that SHH induced gefitinib resistance in xenograft tumors. In contrast, knockdown of GLI1 in gefitinib-resistant PC9 cells resulted in significant reductions in tumor volume and weight, as well as a remarkable increase in TUNEL-positive tumor cells (Fig. 5E-H). This suggesting that depletion of GLI1 could restore the sensitivity of chemoresistant tumors to gefitinib.

### GDC-0449 and gefitinib synergistically increase the sensitivity of tumors to targeted therapy in mice

**Figure 6:**
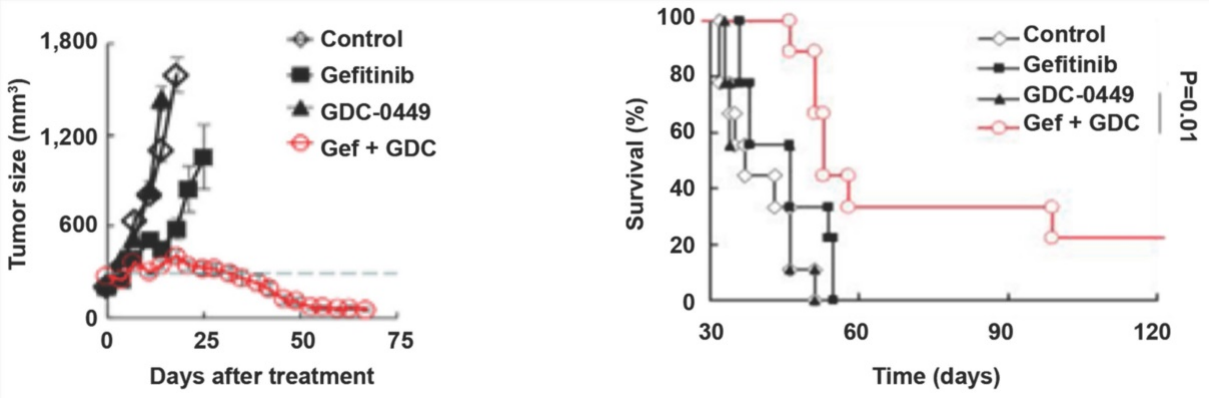
** GDC-0449 and gefitinib synergistically suppress tumor growth and improve the outcomes of tumor-bearing mice.** Mice inoculated with gefitinib-resistant PC9 cells were randomly divided into four groups. After the tumor reached 8 mm in diameter, mice received control, gefitinib (20 mg/kg), GDC-0449 (32 mg/kg), and gefitinib + GDC-0449, respectively, five consecutive days per week for six weeks by gavage. Tumor volumes were recorded in (A). (B) Survival of tumor-bearing mice. Gef, gefitinib.

To evaluate the possible clinical application of Hedgehog pathway suppression in TKI-resistant NSCLC, we established a gefitinib-resistant mouse xenograft model, followed by treatment with GDC-0449 and gefitinib alone or in combination. Compared with GDC-0449 and gefitinib as monotherapies, GDC-0449 and gefitinib in combination considerably reduced the tumor size and prolonged survival time in tumor-bearing mice (Fig. 6A and 6B), suggesting that Hedgehog inhibitor and TKI can synergistically increase the sensitivity of chemoresistant tumors to targeted therapy.

## Discussion

EGFR TKI resistance is a critical clinical challenge in NSCLC therapy. Previous studies have implicated the Hedgehog pathway in chemoresistance for different cancer types. For example, GLI1 activation contributes to doxorubicin, paclitaxel, and cisplatin resistance in breast cancer cells by upregulating the multidrug resistance protein-1 23. SMO gene amplification mediates EGFR TKI resistance in lung cancer by inducing EMT 24. In pancreatic cancer, Hedgehog and EGFR have synergistic effects on ERK and AKT phosphorylation, hence on cell proliferation, survival, and chemoresistance 25. This has also been observed in gastrointestinal, prostate, breast, brain, skin, and liver cancers and leukemia 26-28. Gemcitabine-resistant pancreatic cancer cells display a higher expression of SHH, SMO, and GLI1 in comparison with parental cells 29. In this study collecting data from the TCGA database, we found that lung cancer tissues from EGFR TKI-resistant NSCLC patients had a hyperactive Hedgehog pathway than those from TKI-sensitive counterparts. In NSCLC patients, increased GLI1 expression correlated with prolonged progression-free survival. These findings suggest that Hedgehog signaling induction contributes to EGFR TKI resistance and disease progression in NSCLC.

Furthermore, in NSCLC cases showing brain metastases, tumor tissues from TKI-resistant patients had remarkably increased expression of Hedgehog pathway components compared with those from TKI-sensitive cases. This suggesting that activated Hedgehog signaling also contributes to EGFR TKI resistance in NSCLC cases showing brain metastases. Therefore, the Hedgehog pathway appears to be a therapeutic target for addressing EGFR TKI resistance in NSCLC, including those with brain metastases. However, most of the results have been disappointing 30. Nevertheless, novel strategies aim to inhibit the crosstalk of the Hedgehog pathway with other pathways 31, 32.

A large number of previous studies have discussed the direct impact and potential mechanism of the Hedgehog pathway in a wide variety of human malignancies, including in lung cancer. A previous study on the activation of hedgehog signaling in EGFR TKIs model resistance from EGFR-mutated and wild-type NSCLC revealed that the Hedgehog pathway has a role in mediating resistance to anti-EGFR TKIs via EMT. 33. Another previous study on Hedgehog signaling revealed that it was silenced in EGFR TKI-sensitive NSCLC and inappropriately activated in EGFR TKI-resistant NSCLC. The study implicated that hyperactivity of the Hedgehog signaling caused EGFR TKI resistance, and blockade of this signaling synergistically increases EGFR receptor TKI sensitivity in NSCLC 34. Moreover, a recent study on understanding the molecular mechanisms underlying EGFR-TKI resistance indicated that the miR-506/SHH axis might represent a novel therapeutic target for future EGFR mutated lung cancer treatment 35. In addition, the efficacy of the sequential use of EGFR tyrosine kinase inhibitors and its acquired resistance were investigated, which revealed that it is efficacious in the EGFR-mutant NSCLC 36. Moreover, another study revealed that deregulation of the Hedgehog signaling network might lead to major tissular disorders and the development of cancers, suggesting a pivotal role in the cancer progression 37. Nonetheless, a recent study suggested that the Hedgehog pathway might play a pivotal role in the mediation of pemetrexed resistance in NSCLCs 38. In our study, we have investigated the role of the Hedgehog pathway in association with EMT in EGFR TKI resistance in NSCLC. Our study revealed that the Hedgehog signaling mediates EGFR TKI resistance and induces EMT, suggesting a potential therapeutic target to defeat TKI resistance in NSCLC.

Our results revealed that SHH/GLI1 signaling induction is associated with alterations of EMT markers in NSCLC cells with acquired EGFR TKI resistance. It is well-known that epithelial cells acquire mesenchymal features during EMT by losing cell-cell adhesion features and gaining factors that induce cell migration and invasion 33. N-Cadherin replaces E-Cadherin, enhancing tumor cell motility 3. As shown above, SHH stimulation suppressed E-cadherin and enhanced the migratory and invasive potential of parental PC9 cells, corroborating previously reported findings 34, 35. EMT also reinforces tumor cell resistance to apoptosis by upregulating anti-apoptotic signals 36. Consistently, we found that SHH stimulation inhibited gefitinib-induced apoptosis in parental PC9 cells *in vitro* as well as *in vivo*, suggesting that SHH/GLI1 pathway induction contributes to gefitinib resistance, possibly through EMT-induced apoptosis evasion.

On the other hand, EMT is reversible, with epithelial cells acquiring a mesenchymal phenotype and subsequently returning to a more epithelial phenotype 37. Studies have suggested that the removal of EMT inducers can reverse EMT to enhance the treatment effects of EGFR TKIs in NSCLC 38, 39. Our results demonstrated that knockdown of GLI1 could restore E-cadherin and ZO-1 protein amounts and reduce cell migration and invasion in PC9 cells with gefitinib resistance. Furthermore, GLI1 knockdown in gefitinib-resistant PC9 cells significantly suppressed tumor growth while promoting tumor cell apoptosis, suggesting that depletion of GLI1 increases the sensitivity of chemoresistant tumors to gefitinib, possibly via EMT reversion.

Two hedgehog signaling inhibitors, including vismodegib (GDC-0449) and sonidegib, have been approved by the US Food and Drug Administration for treating advanced and metastatic basal cell carcinoma. However, their clinical benefits are limited to certain cancers 40, 41. Joint suppression of hedgehog signaling, and other oncogenic networks might circumvent drug resistance for improving the antitumor efficacy of hedgehog inhibitors 31, 32. It has been reported that hedgehog signaling suppression synergistically acts with EGFR TKIs in inhibiting different NSCLC cell lines. The SMO inhibitor GDC-0449 or BMS-833923 synergizes with erlotinib to abrogate self-renewal events in stem-like NSCLC cells 42. The SMO antagonist SANT-1 cooperates with gefitinib to inhibit cell growth in NSCLC with EGFR TKI resistance. GDC-0449 administration also shows a synergic effect with cisplatin on chemoresistant NSCLC cells 43. The present study showed that GDC-0449 and gefitinib in combination considerably reduced the tumor size and prolonged the survival of tumor-bearing mice compared with GDC-0449 and gefitinib as monotherapies. This further supports the notion that blocking the hedgehog pathway synergizes with EGFR TKIs to increase the sensitivity of NSCLC chemoresistant to TKIs.

We recruited patients with brain metastasis to study the effect of EGFR-TKI on the cancer cell response. Indeed, EGFR-TKIs are able to cross the blood-brain barrier, while most other anticancer treatments cannot cross it 44. Therefore, in patients taking multiple drugs, the brain metastases should mainly respond to the EGFR-TKI. In addition, no patients underwent resection for primary tumor after recurrence. Those factors might limit the generalizability of the results. Finally, other types of EMTs were not examined in the present study. This will have to be examined in future studies.

Nevertheless, the literature could provide some insights. Zhang et al. 45 showed that there is crosstalk among EMTs induced by Hedgehog, TGF-β, and WNT and that they cooperate to maintain the Snail pathway and the EMT 46. In addition, the TGF-β pathway is able to activate Hedgehog mediators 47, highlighting the complex relationships among those pathways 45. This is probably why the inhibition of the hedgehog alone yielded disappointing results 30.

This study has several limitations. (i) This study used the human lung cancer cell line PC-9 (Ex19del). However, it lacked normal and other cell cancer cells as internal and external controls, which are to be included in future studies on this topic. The results will have to be verified in other cell lines harboring various EGFR mutations. (ii) The sample size of this present study was relatively small and considered a minuscule larger sample size. Thus, the results will have to be confirmed in larger cohorts through further investigations. (iii) Furthermore, figures of this study also lack high-quality elucidations. Therefore, higher magnifications or sharpness or quality elucidations would carefully be arranged in future studies.

In the previous studies on the Hedgehog pathway activation, authors mainly investigated the Hedgehog pathway in chemoresistance for different types of cancer. In contrast, our study investigated this pathway's function and association with EMT in EGFR TKI-resistance in NSCLC. Due to the fact that EGFR mutations are detected in 20%-40% of NSCLC and promote the progression of cancer 5, investigating this pathway and association with EMT in EGFR TKI-resistance in NSCLC has benefits in respect to those previous studies. These include overexpression of Hedgehog signaling-related genes with EGFR TKI-resistance in NSCLC suggesting brain metastases, promotion of PC9 cells motility and attenuating their sensitivity to gefitinib through Hedgehog signaling, overcoming of EGFR TKI-resistance through knockdown of GLI1 in PC9 cells, revealing that the SHH induces or GLI1 knockdown regulates EMT markers which reverse gefitinib resistance in xenograft tumors, and synergism of GDC-0449 and gefitinib increase tumors sensitivity.

Overall, the above findings indicate that Hedgehog signaling mediates EGFR TKI resistance, possibly by inducing EMT. Therefore, blocking the Hedgehog pathway is a potential therapeutic strategy to defeat TKI resistance in NSCLC.

## Supplementary Material

Supplementary tables.

## Figures and Tables

**Table 1:** Characteristics of patients

Characteristics	EGFR TKI-sensitive patients (n = 20)		EGFR TKI-resistant patients (n = 11)	*P* value		
Age (years)				0.879		
≤65	11 (55%)		6 (54.5%)			
>65	9 (45%)		5 (45.5%)			
Gender				0.645		
Female	13 (65%)		7 (63.6%)			
Male	7 (35%)		4 (36.4%)			
Smoker				0.915		
Yes	6 (30%)		3 (27.3%)			
No	14 (70%)		8 (72.7%)			
ECOG PS				0.537		
0-1	9 (45%)		5 (45.5%)			
2	11 (55%)		6 (54.5%)			
Histological subtypes					0.989	
Adenocarcinoma	19 (95%)		11 (100%)			
Squamous cell carcinoma	1 (5%)		0 (0)			
The number of metastatic brain foci					0.909	
<3	4 (20%)	3 (27.3%)				
≥3	16 (80%)	8 (72.7%)				
Extracranial lesion					0.897	
Yes	17 (85%)	10 (90.9%)				
No	3 (15%)	1 (9.1%)				
EGFR mutation					0.999	
Exon 19 deletion	11 (55%)	6 (54.5%)				
Exon 21 L858R mutation	9 (45%)	5 (45.5%)				
Brain symptoms					0.516	
Yes	16 (80%)	9 (81.8%)				
No	4 (20%)	2 (18.2%)				
						

EGFR, epidermal growth factor receptor; TKI, tyrosine kinase inhibitor; ECOG PS, Eastern Cooperative Oncology Group performance status.
