# Supplementary material for: Activation of the Hedgehog pathway mediates resistance to epidermal growth factor receptor inhibitors in non-small cell lung cancer
Source: J Cancer. 2022 Jan 4;13(3):987–97. doi: 10.7150/jca.63410 (PMC8824885; doi:10.7150/jca.63410)
Supplement: Supplementary file 1 — Supplementary tables. [file jcav13p0987s1.pdf]

**Table S1. Small hairpin RNAs**

| Name | Forward（5'-3'） | Reverse primer (5'-3') |
|---|---|---|
| shRNA-GLI1-1 | CCGGTACATCAACTCCGGCCAATAGCTCGAGCTATTGGCCGGAGTTGATGTATTTTTG | AATTCAAAAATACATCAACTCCGGCCAATAGCTCGAGCTATTGGCCGGAGTTGATGTA |
| shRNA-GLI1-2 | CCGGTACCTGCTTCGGGCAAGATATCTCGAGATATCTTGCCCGAAGCAGGTATTTTTG | CCGGTACCTGCTTCGGGCAAGATATCTCGAGATATCTTGCCCGAAGCAGGTATTTTTG |

shRNA, small hairpin RNA; GLI1, glioma-associated oncogene homolog 1.

**Table S2. Gene-specific primers for quantitative real-time PCR**

| Gene | Forward primer（5'-3'） | Reverse primer (5'-3') |
|---|---|---|
| GAPDH | | |
| GLI1 | AGCGTGAGCCTGAATCTGTG | CAGCATGTACTGGGCTTTGAA |
| SMO | GGGTGCCGGAAGTCATACTC | GCTAGGATCTGTATAGCGTTTGG |
| BCL2 | AACGCTATACAGATCCTAGCTCG | GTGCCGTTTGGTCACATGG |
| PTCH1 | GAAGAAGGTGCTAATGTCCTGAC | GTCCCAGACTGTAATTTCGCC |
| HHIP | CCAGAAAGTATATGCACTGGCA | GTGCTCGTACATTTGCTTGGG |
| SNAIL | ACTTCAAGGGGTACGAGTATGT | TGCGACACTCTGATGAACCAC |
| CYCLIND | TCTCAAAGCCTGTTCCACTCA | GCCTCGGCAAGTGTAAAAGAA |

GLI1, glioma-associated oncogene homolog 1; SMO, smoothened; BCL2, B-cell lymphoma 2; PTCH1, patched; HHIP, Hedgehog interacting protein.
